# Mycosis Fungoides in Iranian Population: An Epidemiological and Clinicopathological Study

**DOI:** 10.1155/2015/306543

**Published:** 2015-01-28

**Authors:** Farahnaz Fatemi Naeini, Bahareh Abtahi-Naeini, Hamidreza Sadeghiyan, Mohammad Ali Nilforoushzadeh, Jamshid Najafian, Mohsen Pourazizi

**Affiliations:** ^1^Skin Diseases and Leishmaniasis Research Center, Department of Dermatology, Isfahan University of Medical Sciences, Isfahan, Iran; ^2^Students' Research Committee, Isfahan University of Medical Sciences, Isfahan, Iran; ^3^Skin and Stem Cell Research Center, Tehran University of Medical Sciences, Tehran, Iran; ^4^Cardiovascular Research Center, Cardiovascular Research Institute, Isfahan University of Medical Sciences, Isfahan, Iran; ^5^Students' Research Committee, Semnan University of Medical Sciences, Semnan, Iran

## Abstract

*Background*. Mycosis fungoides (MF) is the most common subtype of cutaneous T-cell lymphoma. Extensive studies on Iranian MF patients are absent. The present study aimed to produce updated clinical information on Iranian MF patients. *Methods*. This was a retrospective, descriptive, single-center study, including all cases of MF seen in the Department of Dermatology, University Hospital of Isfahan, Iran, between 2003 and 2013. Data systematically recorded for each patient included clinical, biological, histological, and molecular findings. *Results*. Eighty-six patients with clinical and histologic diagnosis of MF were included in the study. Thirty-nine patients (45.3%) were male. Female predominance was observed in patients (male : female ratio is 1 : 1.2). Patients were between 7 and 84 years of age (median: 41). The interval from disease onset to diagnosis ranged from 0 to 55 years (median: 1 year). Eighteen cases (20.9%) had unusual variants of MF. The most common types included hypopigmented and poikilodermatous MF. Childhood cases of MF constituted 5.8% (5/86) of all patients. The early stages were seen in 82 cases (95.34%). *Conclusion*. The major differences in epidemiologic characteristics of MF in Iran are the lack of male predominance and the lower age of patients at the time of diagnosis.

## 1. Introduction

Mycosis fungoides (MF) is the most common subtype of cutaneous T-cell lymphoma (CTCL). MF differs from other primary CTCLs by virtue of unique clinical features and histopathology [1, 2]. Patients with MF commonly present with persistent and/or slowly progressive skin lesions of varying size and shape [3].

The natural history of MF is characterized by an indolent progression through four stages: patch, plaque, tumor, and visceral involvement, but this progression is not necessarily seen in all patients [4]. Many variants of this lymphoma substantially differ from “classic” MF and are therefore sometimes referred to as “atypical” forms of the disease [4]. Atypical forms of MF include hypopigmented, hyperpigmented, ichthyosiform, pityriasis lichenoides-like, granulomatous, folliculotropic, bullous, palmoplantar, pagetoid reticulosis, and granulomatous slack skin [3, 4].

The peak age at presentation is in excess of 55 to 60 years, with a 2 : 1 male : female ratio [5, 6]. Although MF is a disease mainly seen in older patients, it can be seen in patients under the age of 35 with similar clinical findings [7, 8].

Extensive studies on Iranian MF patients are absent. The present study thus aimed to produce updated clinical information on Iranian MF patients, including epidemiology, patient characteristics, clinicopathologic features, and treatment.

## 2. Methods

Between 2003 and 2013, patients with MF from single-center resident training hospitals had been retrospectively enrolled into the study.

A diagnosis of MF was confirmed according to International Society for Cutaneous Lymphoma (ISCL)/EORTC criteria [1, 9] and, if necessary, based on the proposed algorithm for early phase disease [10].

The analyzed clinical data included the age of the patients at the time of diagnosis, the age of the patients at the time of initiation, the cutaneous lesion, sex, and the status of disease at the last follow-up examination. Hematoxylin and eosin-stained slides, immunohistochemical stains for basic B- and T-cell markers (CD20 and CD3 and/or CD45RO), and additional immunostaining, including CD4, CD8, CD30, and CD5, were histologically evaluated by an expert dermatopathologist. Polymerase chain reaction analyses of T-cell receptors (TCR) b or c and immunoglobulin heavy chain gene rearrangement were reviewed.

In all the patients, the presence of extracutaneous disease at the time of diagnosis had been excluded by standard staging procedures.

Stage workup (chest radiology, bone marrow biopsy, computed tomography scan of chest, abdomen, and pelvis) and overall survival were obtained by reviewing the clinical records.

To determine the stage of MF, the tumor-node-metastasis (TNM) system was used [11]. The clinical staging was performed as proposed by Olsen et al. [12].

After data collection, statistical analysis was performed by the SPSS software version 19.

## 3. Results

Eighty-six patients with clinical and histologic diagnosis of MF were included in the study.

Table 1 summarizes the demographic profiles and basic data of the 86 patients in our study with MF (Table 1). 45.3% patients (39 cases) were male. Female predominance was observed in the patients (male : female ratio is 1 : 1.2). All patients were Iranian. The patients with MF were between 7 and 84 years of age (median: 41). The mean ± standard deviation (SD) age at the time of skin lesion initiation was 45.02 ± 17.47 and 38.80 ± 16.02 for males and females, respectively.

The interval from disease onset to diagnosis ranged from 0 to 55 years (median: 1 year).

**Figure 1:**
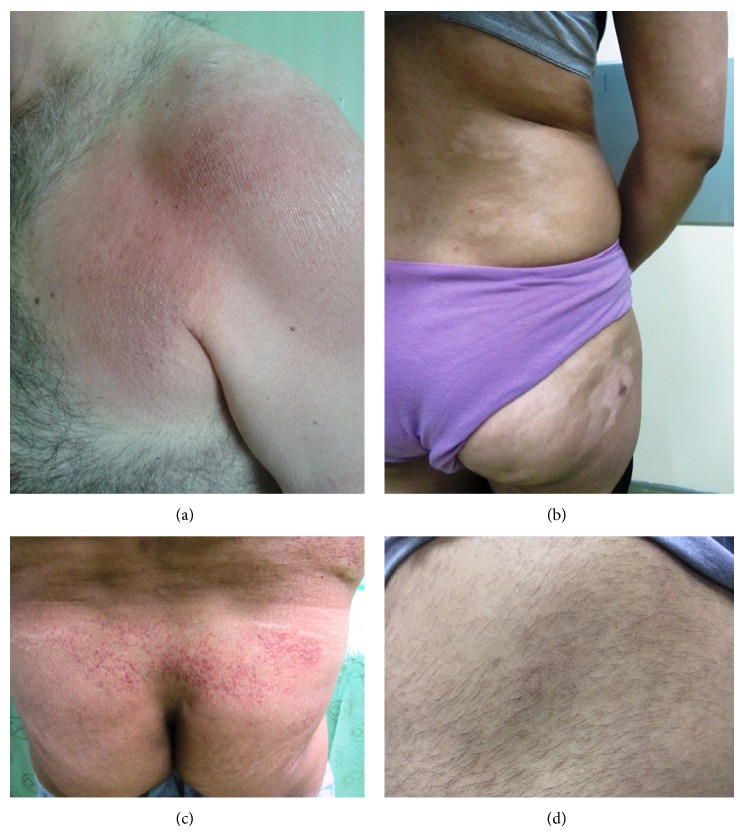
(a) Classic mycosis fungoides. Erythematous mildly scaly patch in the upper trunk. (b) Hypopigmented mycosis fungoides. Multiple hypopigmented and ill-defined to well-defined patches were seen on the back and thigh. (c) Poikilodermatous mycosis fungoides. The lesions are characterized by hypopigmentation and hyperpigmentation with atrophy and telangiectasia. (d) Folliculotropic mycosis fungoides. Diffuse perifollicular inflammatory papules coalescing into plaques.

Table 2 summarizes the clinical and histologic variants of the patients with MF (Table 2). At the time of diagnosis, the most prevalent skin lesions were an erythematous scaly patch (Figure 1).

From 68 patients with classic MF, 42 patients presented with the patch, 21 with plaque, 4 with a tumor, and only one with erythroderma (Table 2). From 86 patients with MF, 18 cases (20.9%) had unusual variants. The most common types included hypopigmented MF (Figure 1(b)) and poikilodermatous MF (Figure 1(c)). Other clinical types of MF including pityriasis lichenoides-like lesions, syringotropic, ichthyosiform, and bullous variant were not observed in our patients. Hypopigmented skin lesions were seen in 5 (5.8%) patients and included a pure hypopigmented variant in three patients, whereas others had hypopigmented lesions mixed with other types. This group included 4 women and one man, 15–34 years of age, at diagnosis (Table 2). The patients with hypopigmented MF were observed to have a younger mean age at diagnosis (25.60 ± 7.30 years) as compared with other MF cases (42.52 ± 16.80 years).

Only one patient had folliculotropic MF (Figure 1(d)). One patient had lymphomatoid papulosis accompanying MF. Three patients (3.5%) had a solitary MF lesion, whereas others had multiple lesions (Table 2).

Childhood cases of MF (younger than 18 years) constituted 5.8% (5/86) of all the MF patients. There were two male and three female patients (male : female ratio is 1 : 1.5).

There was no familial clustering. None of the patients had HIV or other forms of immunosuppression.

According to the TNM classification, among 86 cases of MF, the early stage (stage I + IIA) included 82 cases (95.34%) and the advanced stage (stage IIB + III + IV) included four cases (4.66%). One female patient had a large cell transformation during the course of disease. Progression to the late stages of MF is not seen in childhood/juvenile-onset MF patients.

Immunophenotypic analysis demonstrated that 77.6% of the patients with MF were CD4 positive. CD20 was negative in all patients. In our patients, the conventional T-helper phenotype (CD4+/CD8−) was the most common one (63% of patients) (Table 3).

A clonal T-cell receptor rearrangement was detected in the skin biopsy in 9 patients (36%) from a total of 25 patients in whom T-cell receptor analysis was possible.

Antibodies to HTLV-1 were not detected in the sera of any patients with MF by ELISA. Seven patients (8.1%) had abnormal lactate dehydrogenase (LDH). None of the patients died during the study period in the early stages.

Table 4 summarizes the different treatment modalities used (Table 4). Thirteen patients (15.3%) of the whole achieved complete clearance.

## 4. Discussion

The major differences in the epidemiologic characteristics of MF in Isfahan, Iran, are lack of male predominance and the lower age of patients at the time of diagnosis in those reported from the West. It was reported that the incidence rate of MF in Isfahan is the same as that in other parts of Iran, which is largely similar to the data of other countries [7].

MF is generally rare among Asians and it has a higher incidence rate in blacks [13, 14]; it can be concluded that immunogenetics or interactions of genetic susceptibility and the environment may have a role in MF incidence [5].

There is a male predominance in almost all studies on CTCLs and MF, with a male : female ratio of 1.3 : 1 to 2 : 1 [15–17].

The proportion of our patients complaining on the onset of their disease before 21 years was much higher, similar to the epidemiological study in Kuwait [18]. The occurrence of MF in younger patients in Isfahan, Iran, may be related to some ethnic or genetic variations, but the environmental and occupational factors need to be determined.

Some studies have found an increased incidence of CTCL among workers in chemical science, transportation, and manufacturing industries, whereas other studies have not [19].

Hydrocarbons and petrochemical exposure have been reported to carry an increased MF risk [20, 21]. Whether a greater exposure to chemical agents has some contribution to the course of MF in this region remains to be determined.

In the Iran-Iraq War (1980–1988), it is not known whether chemical weapons by Iraq contained Sulfur mustard (SM), a DNA alkylation, and a well-known carcinogenic agent [22]. This hypothesis can be considered for relatively younger ages of diagnosis among the population in Kuwait. The exposure to burning oil during the war contributed to environmental pollution [18]. To evaluate a possibility of pollutants playing a role in the etiology of CTCL, more data needs to be addressed as potential confounders in future studies.

The prevalence of childhood MF among different studies has varied between 2.7% and 16.6% [23, 24]. In the present study, about 6% of cases were younger than 18 years at the time of diagnosis. Thus, our study is compatible with these reports. There was no significant difference in gender in our study in childhood MF.

Among the atypical forms of MF, hypopigmentation is one of the most prevalent forms in Asians [25]. Hypopigmented MF is overwhelming in Asians, with only 16 cases reported as of 2012 in Caucasians [3]. Compared with other clinical manifestations of MF, the hypopigmented MF is more prevalent in young age groups [18, 25]. The reason for the relative high frequency of hypopigmented MF in young patients is not known [26, 27]. In agreement with our study, the mean age at diagnosis is lower in the hypopigmented compared with the classic variant [28, 29]. On the other hand, the hypopigmented variant was noted in 79% of patients less than 18 years old [30].

Our data is compatible with the study of Quaglino et al. [31] in the fact that the current study showed a minority of patients were found to have tumor lesions or erythroderma and extracutaneous involvement. The definitive diagnosis of MF, particularly early stage of disease, is challenging as many of its clinical and pathologic features are nonspecific.

The median time from symptom onset to diagnosis in retrospective studies is 3-4 years, but it may exceed four decades [14, 32, 33].

The current study differs from that of Quaglino et al. in the fact that the median time from the onset of cutaneous lesions to diagnosis was 2 years [31].

In the present study, the mean time from symptom onset to definite diagnosis was about 5–10 years, which is significantly different in male and female patients.

Several studies have shown that nearly all patients with MF are HTLV-1 seronegative [34]. Our findings do not support an association between HTLV-1 infections and showed that MF does not correlate with HTLV-1 infections in Isfahan, Iran.

Usually, MF is characterized by an infiltrate of T-helper memory lymphocytes (CD3+, CD4+, CD5+, CD8−, and CD45RO+) [1]. However, in a minority of cases the neoplastic cells exhibit a T-cytotoxic (CD4−/CD8+) or a CD4/CD8 double-negative phenotype that shows no clinical and/or prognostic differences [35]. de Marchi et al. showed that the coexpression of CD4 and CD8 in patients with MF is associated with a slightly lower rate of progressive disease compared with patients with conventional CD4+/CD8− phenotype. These findings raise the possibility that the coexpression of CD4 and CD8 in cutaneous lesions may confer a better prognosis in MF [36]. The lower tendency of progression of the disease in our patients with the coexpression of CD4 and CD8 might be related to the increased activity of antitumor CD8+ lymphocytes infiltrating the lesions [36].

## 5. Conclusions

Although the group of this study is really not very large and also the number of the patients with more advanced disease is small, it may be the starting point for a larger multicenter study in Iran. Whether the occurrence of MF in younger patients in Iran is related to some ethnic or genetic variations or to environmental factors needs to be determined. A prospective multicenter study on a larger population of patients with a longer follow-up might be useful to confirm the possible causes of these epidemiological differences.

## Figures and Tables

**Table 1:** Demographic profile and basic data of 86 patients with mycosis fungoides.

Age (years)	
Range	7–84
Median	41
Mean (±SD)	41.51 ± 16.85
Sex	
Male	39 (45.3%)
Female	47 (54.7%)
Latent period (years)	
Range	0–55
Median	1
Mean (±SD)	4.67 ± 8.70
Type of MF	
Classic	68 (79.06%)
Variant	18 (20.94%)
TNM stage	
IA	36 (41.9%)
IB	43 (50%)
IIA	1 (1.2%)
IIB	5 (5.8%)
IIIA	1 (1.2%)
IVA1	0
IVA2	0
IVB	0

**Table 2:** Clinical characteristics of patients based on the different clinical variants of mycosis fungoides.

Classification	Number of cases (%)	Sex		Age, year median (range)	Latent period, year median (range)	Staging	
		M	F			Early	Advanced
Classic type of MF (*n* = 68)							
(i) Patch	42 (48.8)	19	23	42 (7–82)	1 (0–27)	42	0
(ii) Plaque	21 (24.4)	11	10	48 (19–84)	1 (0–30)	21	0
(iii) Tumoral	4 (4.7)	3	1	55 (31–59)	7 (3–25)	2	2
(iv) Generalized erythroderma	1 (1.2)	1	0	45	1	0	1

Variant type of MF (*n* = 18)							
(i) Hypopigmented	5 (5.8)	1	4	28 (15–34)	3 (1–10)	5	0
(ii) Poikilodermic	5 (5.8)	2	3	44 (23–62)	1 (0–55)	5	0
(iii) Solitary	3 (3.5)	2	1	31 (30–38)	0 (0–6)	3	0
(iv) Hyperpigmented	2 (2.3)	0	2	32 (23–41)	0	2	0
(v) Folliculotropic	1 (1.2)	0	1	45	1	0	1
(vi) Pigmented purpura-like lesion	1 (1.2)	0	1	26	0	1	0
(vii) Hyperkeratotic	1 (1.2)	0	1	18	0	1	0

**Table 3:** T-helper phenotype in 86 Iranian patients.

T-helper phenotype	Frequency	Percent (%)
CD4+/CD8+	13	15.5
CD4−/CD8−	4	4
CD4+/CD8−	54	63
CD4−/CD8+	15	17.5

**Table 4:** Treatment modalities used for mycosis fungoides in 86 Iranian patients^a^.

Treatment modality	Number (%)
Topical corticosteroids	7 (8.1)
Topical carmustine (BCNU)	37 (43)
Topical nitrogen mustard	1 (1.2)
Narrow-band UVB (NBUVB)	33 (38.4)
Oral psoralen plus UVA (PUVA)	23 (26.7)
Local radiotherapy	3 (3.5)
Chemotherapy (systemic)	1 (1.2)
Methotrexate	1 (1.2)
Interferon	3 (3.5)

^a^Many patients had more than one treatment modality at different periods of their disease.
